# Trends in Smoldering Myeloma Incidence in the United States From Cancer Registries, 2012–2022

**DOI:** 10.1002/ajh.70202

**Published:** 2026-01-13

**Authors:** Rong Wang, Amy J. Davidoff, Martin Schoen, John H. Huber, Eric J. Feuer, Jennifer Ruhl, Natalia Neparidze, Xiaomei Ma, Su-Hsin Chang, Shi-Yi Wang

**Affiliations:** 1 Department of Chronic Disease Epidemiology, Yale School of Public Health, New Haven, Connecticut, USA; 2 Cancer Outcomes, Public Policy, and Effectiveness Research (COPPER) Center, Yale University, New Haven, Connecticut, USA; 3 Division of Cancer Control and Population Sciences, National Cancer Institute, Bethesda, Maryland, USA; 4 Division of Hematology and Oncology, St. Louis University School of Medicine, St. Louis, Missouri, USA; 5 Division of Public Health Sciences, Department of Surgery, Washington University in St. Louis School of Medicine, St. Louis, Missouri, USA; 6 Department of Internal Medicine, Section of Hematology, Yale School of Medicine, New Haven, Connecticut, USA

## To the Editor,

Smoldering multiple myeloma (SMM) is an asymptomatic plasma cell proliferative disorder, and patients with SMM have a 10% annual risk of progression to multiple myeloma (MM) in the first 5 years after diagnosis [1]. Analyzing data from the Swedish Myeloma Registry, researchers reported that the age-standardized incidence of SMM was 0.44 cases per 100 000 person-years in 2008–2011 [2]. In the United States (US), the Surveillance, Epidemiology, and End Results (SEER) Program did not release separate data of SMM and symptomatic MM until April 16, 2025. To our knowledge, only one US study used hospital registry data to identify SMM using a diagnosis of MM without any treatment recommendation within 120 days after diagnosis and reported an incidence of 0.9 cases per 100 000 person-years during 2003–2011 [3]. Using the newly released cancer registry data on SMM, this study provided updated estimates of SMM incidence.

We identified patients diagnosed with MM from 2012 to 2022 in SEER using the International Classification of Diseases for Oncology, 3rd edition, code 9732. Applying the SEER’s MM site-specific variable [4], which was derived from pathology reports and clinician’s statements [5] and has been available in the newly released SEER databases, we categorized overall MM into symptomatic MM, SMM, and other/unknown. Incidence rates and their corresponding 95% confidence intervals (CIs) were calculated per 100 000 person-years and age-adjusted to the 2000 US standard population. Given the COVID pandemic, we estimated delay-adjusted rates on the incidence of overall MM during 2020–2022 (SMM specific delay-adjustment factors are not available) [6]. To examine trends, we performed joinpoint regression analyses to estimate annual percentage changes, excluding Year 2020. All analyses were conducted using SEER*Stat (Version 8.4.5) and Joinpoint Regression Program (Version 5.4.0.0) (see details in Supporting Information). No ethical clearance was required as all data are publicly available from http://seer.cancer.gov.

**FIGURE 1 |:**
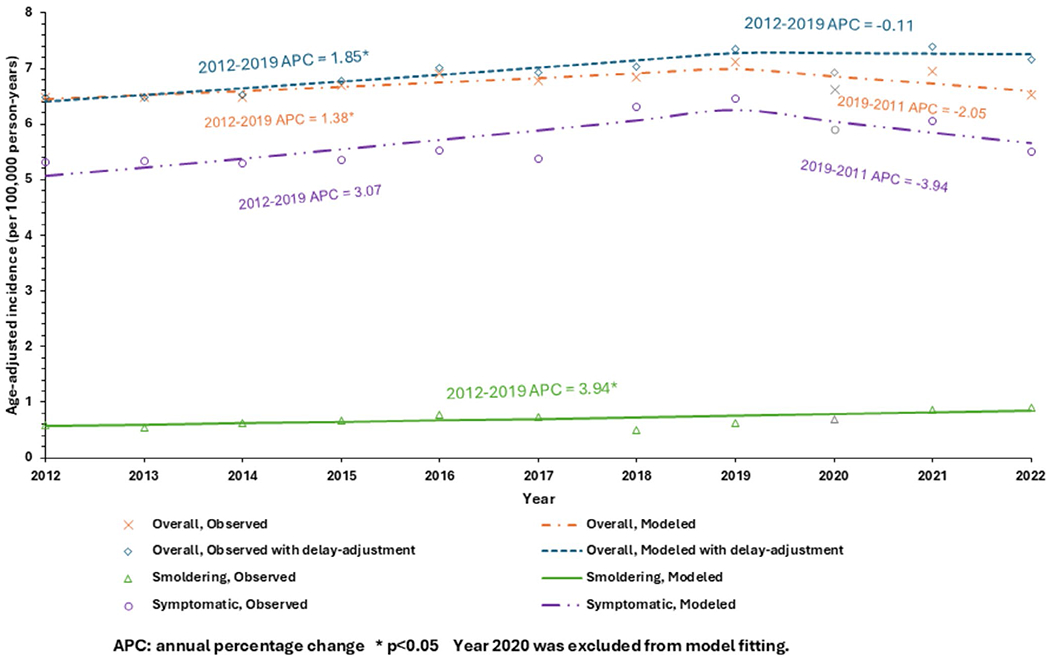
Incidence of overall multiple myeloma, symptomatic multiple myeloma, and smoldering multiple myeloma by year, 2012–2022, SEER 17.

During 2012–2022, the observed incidence of overall MM was 6.72 (95% CI 6.67–6.77) per 100 000 person-years (Figure 1). This represents a significant increase during 2012–2019 (*p* = 0.03), followed by a non-significant decrease during 2019–2022 (*p* = 0.08). The results of delayed-adjusted incidence of overall MM were similar. The mean incidence of SMM was 0.69 (95% CI 0.67–0.70) per 100 000 person-years, with a steady increase from 0.59 in 2012 to 0.90 per 100 000 person-years in 2022 (*p* = 0.01).

**FIGURE 2 |:**
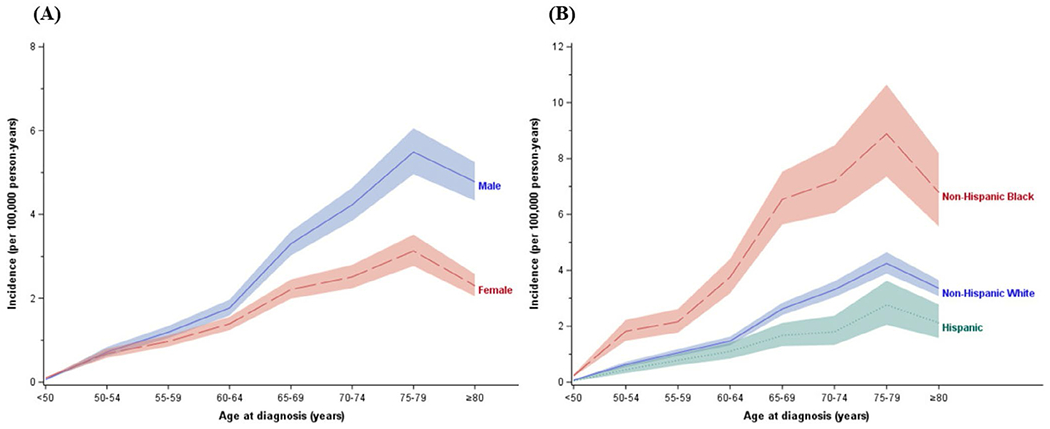
Age-specific incidence of smoldering multiple myeloma, SEER 17, 2012–2019: (A) by sex; (B) by race/ethnicity.

The incidence of SMM increased with age through 75–79 years but decreased after age 80. Males had higher SMM incidence than females, especially after age 60 (Figure 2A). Non-Hispanic Black (NHB) populations had higher SMM incidence across all age groups, compared to Non-Hispanic White (NHW) or Hispanic populations (Figure 2B).

Our study shows the incidence of SMM to be 0.69 per 100 000 person-years with a significant increase of 50% during 2012–2022. This rise may be primarily attributable to change in administrative reporting and diagnosis criteria, as well as evolving clinical management of SMM. Moreover, the growing prevalence of obesity and the increased cumulative environmental/occupational exposure to pollution and pesticides/herbicides may also play a role. Last, the incidence of SMM is much lower than that of symptomatic MM and the absolute change of SMM incidence during the 11-year period is low (0.31 per 100 000 person-year); therefore,a small change in the incidence contributes to a high percentage increase. The observed higher incidence of SMM in NHB/male than NHW/female populations is consistent with NHB race and male sex being risk factors for monoclonal gammopathy of undetermined significance and MM. Similarly, the iSTOPMM study reported higher prevalence of SMM in males (0.67%) than that in females (0.39%) [7].

Our study is the first study to report the SMM incidence in the US using data from cancer registries. Nonetheless, several limitations should be acknowledged. First, registry data lack the granular laboratory and clinical detail necessary to establish a definitive SMM diagnosis. Therefore, we relied on the MM site-specific variable [4] to identify patients with SMM, which was derived from pathology reports and clinician’s statements [5]. Second, SMM is often undiagnosed/under-reported due to its asymptomatic nature and no definitely recommended therapy for SMM. Given that our SMM incidence estimate is based on reported cases, the incidence estimate would increase when including undiagnosed SMM patients. For reference, a national screening study in Iceland reported that the prevalence of SMM was 0.53% in the Icelandic population aged ≥ 40 years [7]. Given the ongoing debates regarding the optimal timing to treat MM, our results could provide important insight when evaluating the impact of SMM management.

## Supplementary Material

Supporting information

Additional supporting information can be found online in the Supporting Information section. **Data S1:** ajh70202-sup-0001-Supinfo.docx.

## Data Availability

All data are publicly available from http://seer.cancer.gov.
