# Supplementary material for: Trends in Smoldering Myeloma Incidence in the United States From Cancer Registries, 2012–2022
Source: Am J Hematol. 2026 Jan 13;101(4):923–5. doi: 10.1002/ajh.70202 (PMC12912868; doi:10.1002/ajh.70202)
Supplement: Supplementary file 1 — Data S1: ajh70202‐sup‐0001‐Supinfo.docx. [file AJH-101-923-s001.docx]

**Supplement: Detailed Methodology**

We employed Incidence - SEER Research Plus Data from the National Cancer Institute (NCI)'s Surveillance, Epidemiology, and End Results Program SEER*Stat Database, 17 registries, November 2024 submission. SEER 17 encompasses approximately 26.5% of the US population.^1^ All data are from publicly available SEER database of the NCI (<http://seer.cancer.gov>); therefore, no ethical clearance is required for this study.

We included patients diagnosed with MM between 2012 and 2022, identified by the International Classification of Diseases for Oncology (3rd edition) code, 9732. Only patients with malignant cases with known age were included. For Research and Research Plus databases, in the April 2025 release,^2^ SEER*stat introduced a new variable named “Multiple Myeloma Terminology Recode (2012+)”, which is available for patients classified as plasma cell myeloma in both the Tumor-Node-Metastasis (TNM) cancer staging (7th edition)/Collaborative Staging Schema (version 0204+) & extent of Disease (EOD) Schema Id from 2012. The variable consists of three distinct values: “Multiple/Non-secret/Plasma cell/Ultra-High-Risk Smoldering Multiple/NOS Myeloma”, “Smoldering plasma cell/Asymptomatic plasma cell/Early/Evolving myeloma”, and “Other terminology describing myeloma; Unknown terminology used”.^3^ We defined “Smoldering Plasma Cell/Asymptomatic Plasma Cell/Early/Evolving Myeloma” as smoldering MM.

Incidence rates per 100,000 person-years were calculated and age-adjusted to the 2000 U.S. standard population. The corresponding 95% confidence intervals (CIs) for age-adjusted incidences were calculated using Tiwari et al.’s approach.^4^ We also applied delay-adjusted rates in consideration of report delay.^5^ To examine incidence trends, we performed joinpoint regression analyses to estimate annual percentage change (APC). The joinpoint regression analysis identifies statistically significant changes in trend by determining the best-fitting points for change with up to three joinpoints were allowed in the models.^6^ Since joinpoint regression models do not account for a one-year anomaly in data nor to estimate a change in one single calendar year, we excluded incidences in 2020, the year of COVID-19 pandemic, from trend estimation.^7^ All analyses were conducted using SEER*Stat (Version 8.4.5) and Joinpoint Regression Program, (Version 5.4.0.0), both of which are products of the Surveillance Research Program at NCI.

**Reference**

1. Number of Persons by Race and Hispanic Ethnicity for SEER Participants (2020 Census Data). Available at https://seer.cancer.gov/registries/data.html.
2. National Cancer Institute. SEER Data Change History, April 2025 Release. Available at https://seer.cancer.gov/data/data-changes.html.
3. National Cancer Institute. Site-Specific Data Item Recodes. Available at https://seer.cancer.gov/seerstat/variables/seer/ssdi/.
4. Tiwari RC, Clegg LX, Zou Z. Efficient interval estimation for age-adjusted cancer rates. Stat Methods Med Res 2006; 15(6):547-69.
5. National Cancer Institute. Delay-Adjusted Rates in SEER*Stat. Available at https://surveillance.cancer.gov/delay/rates.html. Assessed on April 26, 2025.
6. Kim HJ, Fay MP, Feuer EJ, Midthune DN. Permutation tests for joinpoint regression with applications to cancer rates. Stat Med 2000;19:335-51 (Erratum in Stat Med 2001; 20:655).
7. National Cancer Institute. Impact of COVID on the April 2023 SEER Data Release. Available at https://seer.cancer.gov/data/covid-impact-apr2023.html. Assessed on April 26, 2025.
